# Reconstruction of a Severe Open Distal Humerus Fracture With Complete Loss of Medial Column by Using a Free Fibular Osteocutaneous Graft

**Published:** 2008-04-29

**Authors:** George K Kouvidis, Byron E Chalidis, Mark I Liddington, Peter V Giannoudis

**Affiliations:** Academic Department of Trauma and Orthopaedics, School of Medicine, University of Leeds, Leeds, United Kingdom;; Department of Plastic Surgery, Leeds General Infirmary, Leeds, United Kingdom

## Abstract

**Objective:** Open intra-articular fractures of the distal humerus can be associated with considerable bone loss and extensive soft tissue damage. The management of these injuries is quite challenging, and the restoration of elbow anatomy may require multiple bone and soft tissue surgical procedures. The purpose of this case report is to present the option of addressing at the same time a complex skin, muscular, and bone distal humerus defect by using a composite vascularized graft. **Methods:** We present a case of a high-energy open fracture of the distal humerus in a polytrauma young patient. Apart from the significant damage of all skin layers and underlying muscle units at the posterolateral side of the elbow, the medial column of the distal humerus (6 cm in length) was completely absent. After patient resuscitation and wound debridement, a free vascularized osteocutaneous fibular graft was used for the reconstruction of the bone defect and the restoration of elbow anatomy. **Results:** No complications were encountered during the postoperative period, and both bone and soft tissues progressed to sound healing. At 26 months follow-up, the patient had a functional and stable elbow and the Mayo Elbow Performance Score was 85 points, which is equivalent to a good result. **Conclusions:** Vascularized osteocutaneous fibular graft could effectively address complex traumatic defects of the elbow joint and enhance the potential for bone healing and early functional recovery.

Open intra-articular fractures of the distal humerus are frequently the result of a high-energy trauma. These injuries can be associated with severe bone and articular cartilage fragmentation, extensive soft tissue damage, and concomitant injuries, which may jeopardize limb integrity and patient survival. Because of the complexity of injury, treatment is generally difficult, and subsequent clinical outcome is often poor.^1^^,^^2^ A case of complete medial column bone loss of the distal humerus after an open elbow fracture and the final outcome after reconstruction via a free vascularized osteocutaneous fibular graft are presented.

## CASE REPORT

**Figure 1:**
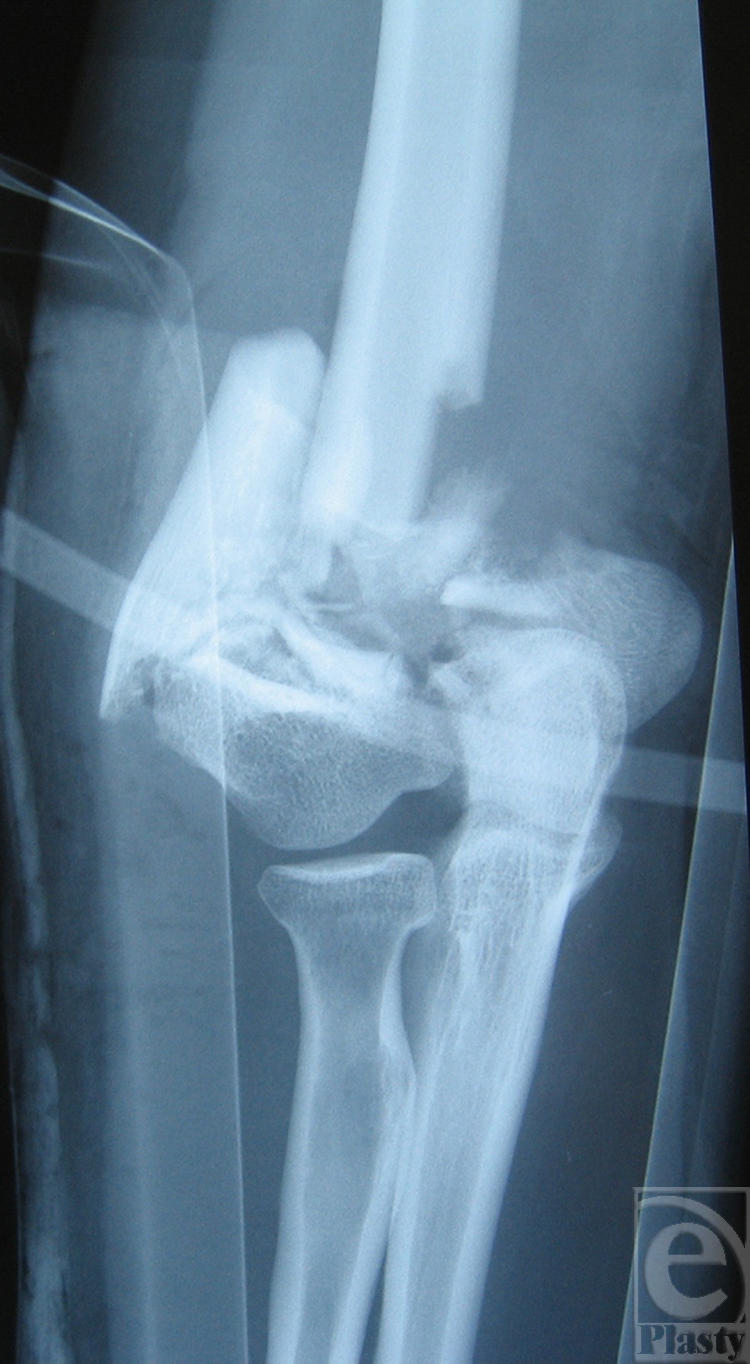
Anteroposterior radiograph of the right elbow. An AO/OTA type C2.3 distal humeral fracture is shown. Except from fracture comminution, bone loss is evident at the medial column of the humerus.

A 24-year-old woman presented to the accident and emergency department of the local hospital after a roadway accident and subsequent fall from a height of approximately 12 m. The patient was alert and orientated, hemodynamically stable, and she was resuscitated according to the standard Advanced Trauma Life Support protocol. The clinical and radiologic examination revealed (1) a right nondisplaced occipital condyle fracture, which was treated with a hard collar; (2) a left-sided renal injury without gross hematuria or adverse effects, which was managed nonoperatively; (3) A pelvic injury with right superior and inferior pubic rami fractures, right sacroiliac joint disruption, and left intraforaminal sacral fracture; and (4) an open distal humeral fracture of the right elbow with associated bone loss. The fracture was classified as Gustilo^3^^,^^4^ grade IIIb and the *Arbeitsgemeinschaft für Osteosynthesefragen* Orthopaedic Trauma Association (AO/OTA)^5^ types C2.3 according to the relevant classification systems for the open and distal humeral fractures, respectively (Fig 1).

**Figure 2:**
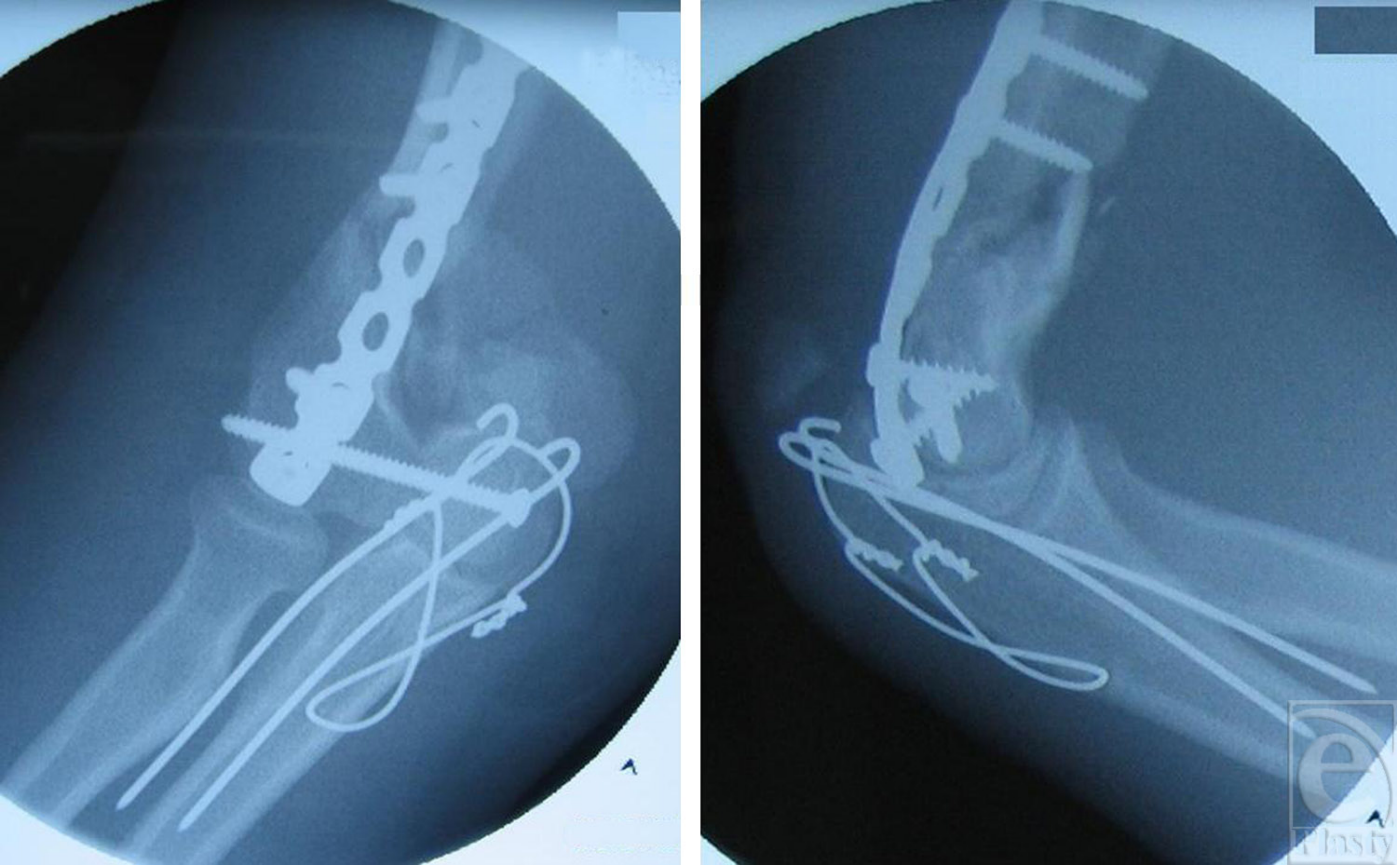
Intraoperative anteroposterior (left) and lateral (right) radiographs of the right elbow after initial fixation of the lateral column and trochlea of the distal humerus. The medial column is completely absent and subsequent reconstruction is unfeasible.

The patient was promptly taken to the operation theatre for stabilization of the right elbow fracture and pelvic ring injury. The elbow wound was debrided and irrigated with 6 L of Hartmann's solution. Removal of all contaminated or dead tissues revealed a large soft tissue defect of approximately 9 × 4 cm in size, which was located at the posterolateral aspect of the elbow and extended until the ulnohumeral joint space. Furthermore, a missing piece of bone—6 cm in length—from the medial humeral column was identified. At this stage, only the trochlear and lateral column fractures were stabilized with a 3.5-mm reconstruction plate and screws, using a transolecranon approach. The wound was temporarily covered and an above-elbow cast was applied (Fig 2). The pelvic injury was addressed with an external fixator and the following day, the patient was referred to our tertiarycare center for further evaluation and management of both elbow and pelvic fractures.

Because of the above findings, a free vascularized osteocutaneous fibular flap for the reconstruction of the right elbow was selected. The procedure was performed 4 days later as patient's resuscitation was optimized to allow reoperation and elbow wound was free of infection or excessive drainage. The flap was harvested using the standard technique as previously described by Chen and Yan.^6^ Following ulnar nerve anterior submuscular transposition, a 6-cm fibular graft was inserted into the medial column defect and subsequently stabilized with a new 3.5-mm reconstruction plate. Microvascular anastomoses of the donor peroneal artery and its accompanying veins were carried out on the corresponding radial artery and venae comitantes after passage of the pedicle through brachialis muscle. On completion of the anastomoses, there was brisk arterial bleeding from the muscle sleeve with satisfactory venous return along venae comitantes. The donor site was closed using a thigh skin graft by the same plastic surgeons' team. Under the same anesthesia and after completion of elbow reconstruction, sacroiliac screw insertion for fixation of pelvic fracture was done. No intraoperative complications were encountered during the 2 surgical procedures.

Postoperatively, a posterior elbow splint was applied and antibiotic medication (cefuroxime, 1.5 g 3 times per day) was continued for 72 hours. The splint was removed periodically on a daily basis, and gentle active elbow flexion-extension exercises were immediately commenced to prevent adhesions and joint stiffness. However, after 10 days, more than 50% of the skin paddle was no longer viable. Debridement of the necrotic skin area was followed by a vacuum negative pressure wound therapy and the skin defect was reconstructed 1 week later with a free anterolateral thigh flap. Finally, the patient was discharged 30 days after the first admission, showing good recovery of her injuries, while instructions to visit the dressing clinic at regular intervals were also given. She was encouraged to continue elbow physical therapy, but no further elbow immobilization was deemed necessary. Evaluation of elbow function and plain elbow radiographs were asked to be done on monthly basis, until bone union was achieved and every 6 months thereafter for the first 3 years.

**Figure 3:**
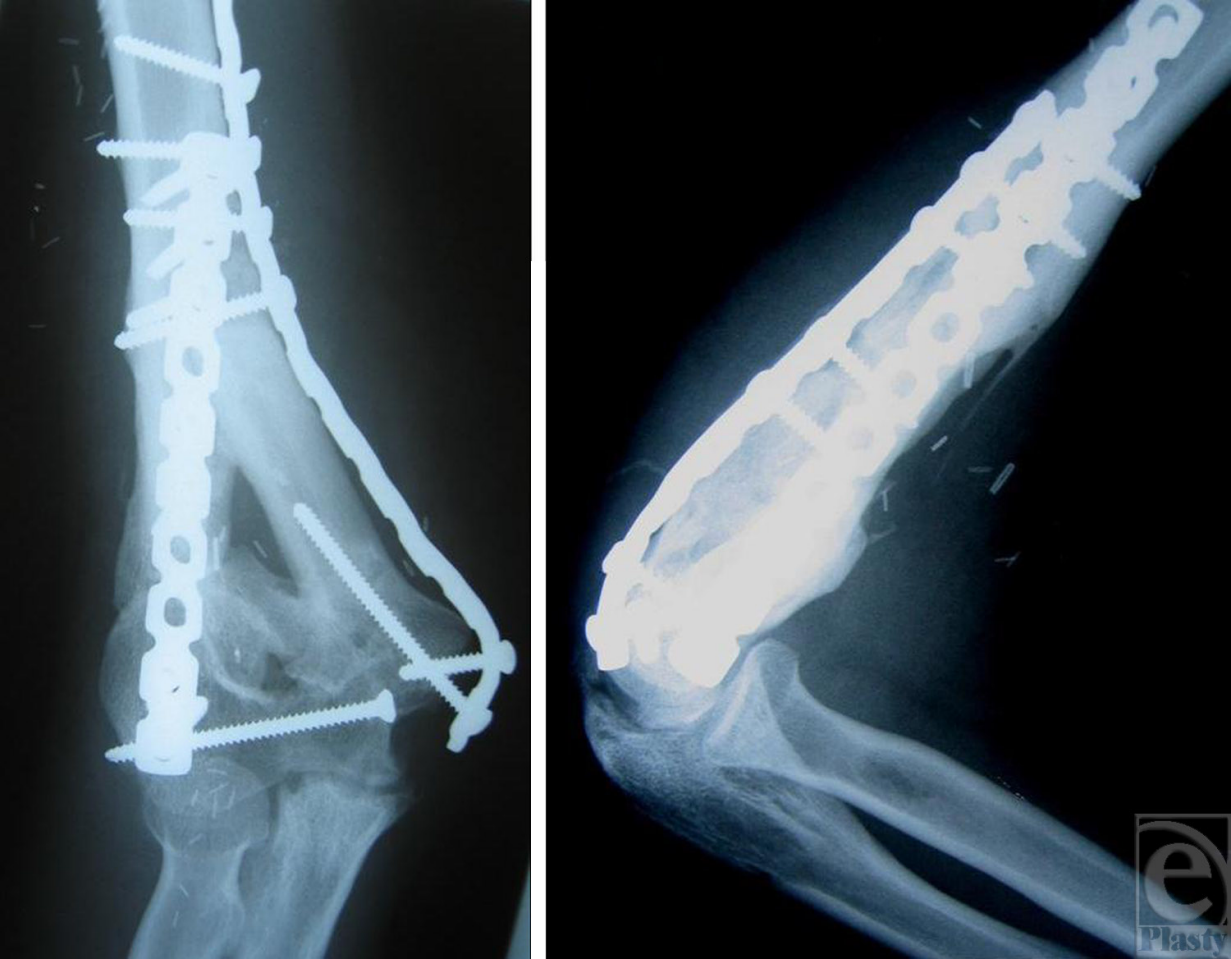
Anteroposterior (left) and lateral (right) radiographs of the right elbow 26 months after transplantation of a 6-cm free vascularized fibular graft. Complete incorporation of the graft and fracture healing are visible.

**Figure 4:**
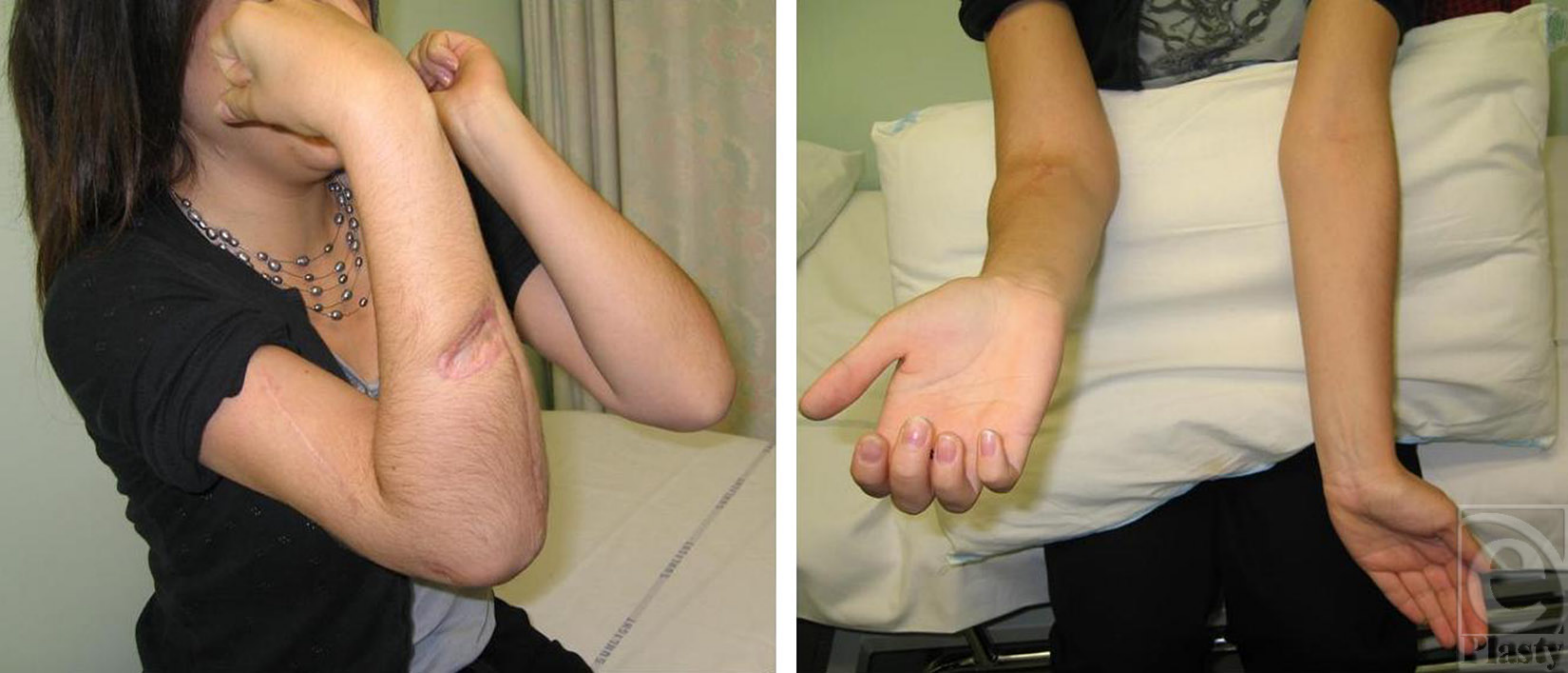
Patient's elbow at 26 months postoperatively. The skin was healed nicely and the patient returned to full mobility. The elbow motion was satisfactory despite the 20° of extension lag (right).

At the final follow-up (26 months), complete incorporation of the bone graft and solid fracture union were evident (Fig 3). Elbow flexion-extension and supination-pronation arcs were 85° and 120°, respectively and a 20° extension lag was apparent. The appearance of skin was satisfactory, and no pain or discomfort was reported. The patient was able to perform her daily activities and returned to her prior-to-injury occupation. No donor side effects were reported and patient's ambulatory status was not compromised at all. The Mayo Elbow Performance Score^7^ was 85 points, which was equivalent to a good result (Fig 4).

## DISCUSSION

Management of bone defects after severe open fractures of the distal humerus encompasses many technical difficulties. In these cases, appropriate osteosynthesis may not be always feasible and bone grafting should be considered for the restoration of normal elbow anatomy.^8^ Vascularized bone transfers are more efficient than conventional corticocancellous interposition grafting for the management of massive bone loss (>6 cm).^9^^,^^10^ They retain their intrinsic blood supply and viability, and the healing process occurs by fracture union rather than “creeping substitution.”^11^ As a result, faster incorporation of the graft and higher union rates should be anticipated.

Nonvascularized conventional bone autografts or bulk allografts constitute a different approach for the reconstruction of distal humerus bone defects.^12^ Their application is quite straightforward, and it does not necessitate a technical expertise or experience. At the same time, a soft tissue procedure such as the transposition lateral arm flap can be performed for the management of soft tissue damage. This flap provides coverage that is thin and supple and promotes the free gliding of the underlying structures.^12^ However, avascular grafts are more susceptible to infection or nonunion as they are probably never replaced by completely healthy bone tissue, and exist as a mixture of necrotic and viable bone with reduced strength.^11^^,^^13^

Limb shortening and cement spacer have also been suggested as alternative solutions for the management of distal humerus bone defects.^14^^,^^15^ In the current case, limb shortening of 6 cm would adversely influence the tone of the muscles and effective moment arm of the muscles causing severe disability in a 24-year-old-woman. On the other hand, cement spacers may retain the soft tissue tension and fill adequately the postinjury defect, but no biologic incorporation or long-term durability should be anticipated. The antibacterial efficacy of bone cement spacers loaded with different combinations of antibiotics makes them a good temporary or permanent treatment choice when severe wound contamination or infection is apparent.

The free osteocutaneous fibula flap is a composite tissue transfer suitable to address combined defects in 1-stage procedure.^16^ The fibula is a long and straight tubular bone, which is not difficult to harvest, while donor site morbidity is minimal up to a graft length of 20 cm.^16^^,^^17^ The anatomy is predictable, and its size and shape allow satisfactory fixation of femoral, tibial, and humeral defects.^16^^,^^17^ It is available with a skin paddle and/or muscle flap to cover soft tissue defects.^18^ Clinical monitoring and early detection of anastomoses failure can be done within hours after the surgery by evaluating the skin paddle viability.^19^ In the herein case, the interposed vascularized graft completed the triangle formed by the lateral and medial bony columns and the trochlea, and also promoted fracture healing and patient recovery.In conclusion, the free osteocutaneous fibular graft should be further considered as a reconstructive option for the treatment of metaphyseal or juxta-articular complex defects of the elbow joint. A combined orthopaedic and plastic surgical management is essential for the appropriate treatment of such cases offering optimum clinical and functional result.
